# Smoking and Depression among Medical School Students: A Cross-Sectional Study from Turkey’s Largest Province †

**DOI:** 10.3390/healthcare12111130

**Published:** 2024-05-31

**Authors:** Bahar Ürün Ünal, Kamile Marakoğlu

**Affiliations:** Department of Family Medicine, Faculty of Medicine, Selcuk University, Konya 42130, Turkey; kmarakoglu@yahoo.com

**Keywords:** medical students, smoking, depression, Beck Depression Inventory, risk factors

## Abstract

Background: To examine the prevalence of smoking among medical faculty students in Turkey, and to explore the associations between smoking, depression, and other factors. Methods: This cross-sectional study was carried out among medical students in Konya, Turkey, from November 2018 to February 2019. The first section included eight questions pertaining to sociodemographic details. The second comprised nine questions addressing smoking and other harmful habits. The third section involved the Fagerstrom Test for Nicotine Dependence while the fourth was the Beck Depression Inventory (BDI). Results: The study was completed with a total of 1117 participants (90.2% of all students). In regard to smoking, 813 (72.78%) were non-smokers, 98 (8.77%) were ex-smokers, and 222 (19.87%) were active smokers. Notably, 16.29% of students (*n* = 182) had a high BDI score (≥17). Male sex, good economic status, depression diagnosis at any time in life, and alcohol use were independently associated with active smoking. Being a senior student and regular exercise were independently associated with a low (<17) BDI score, whereas depression diagnosis at any time in life and drug use were independently associated with high (≥17) BDI. Conclusions: Almost 20% of medical school students were active smokers, with about a 2.5-fold higher prevalence among males compared to females. There is a significant association between smoking frequency and symptoms of depression. Policies targeting modifiable risk factors can reduce smoking and depression among future physicians, which can have a strong impact on population-wide smoking.

## 1. Introduction

Deaths and disabilities due to tobacco use are among the greatest health problems of our time. Tobacco products are the cause of approximately 98% of lung cancer deaths and more than 80% of chronic obstructive pulmonary disease cases. Despite the well-known harmful effects of tobacco products, their use remains widespread throughout the world [1]. The World Health Organization (WHO) reported that the number of tobacco users was 1.32 billion in 2015 and 1.30 billion in 2020, and this number is expected to be at around 1.27 billion by 2025 [2]. Although the marginal decrease is promising, it does not change the fact that currently 8.7 million people die every year due to tobacco use [3]. Approximately 80% of the world’s 1.3 billion tobacco users live in middle- and low-income countries, and in Turkey, a developing country, the proportion of tobacco product users is 30.7% according to WHO 2020 data (42.1% for males and 19.2% for females) [3].

Cigarettes are the most widely used tobacco product worldwide [1]. In order to effectively reduce smoking rates, it is important to raise public awareness about the harms of smoking. This important goal can be achieved with the faithful, effective, and continuous support of healthcare professionals. Studies have found that the countries where smoking rates have been reduced most successfully are the countries with the lowest smoking rates among physicians [4,5]. Medical professionals should persuade their patients to quit smoking, and they should influence their own environment and be role models to prevent the onset of this harmful habit [1,6]. When smoking physicians try to persuade their patients to quit, the intervention is unlikely to be persuasive. Additionally, physicians themselves must be protected from this harmful habit. In fact, some decision- and/or policy makers in health professions have suggested that the smoking status of applicants should be taken into account during enrollment [1]. The ethics surrounding this suggestion may be subject to debate, but this viewpoint emphasizes the importance of fostering healthy lifestyles among physicians. Therefore, it is crucial to take precautions aimed at preventing medical students from smoking and encouraging cessation. Specifically, a vital step involves identifying the factors that prompt medical school students to take up smoking. However, the fact that medical education is a challenging, stressful, and long process may lead to increased smoking among medical students. Although studies have shown lower smoking prevalence among medical students compared to the general population, smoking remains a widespread risk among physicians and medical students [7,8,9,10], particularly since smoking is most commonly initiated soon after high school graduation [1]. The smoking rates among medical students vary depending on a countries’ health policies and cultural and faith-related factors, but can be as high as 50% [7]. Active smoking rates among medical students are higher in Europe than in Asia, and among male students in Turkey than in Europe, which is similar to data from Japan [1]. In some studies, no significant differences have been found between medical and non-medical students in terms of smoking prevalence [7,8,10].

Various studies have expressed that medical school education may be a provoking factor for mental disorders, especially depression, and that depression is a factor that can lead to smoking behavior [11,12,13]. It is necessary to raise awareness among medical faculty students, who are the physicians of the future, about the harms of smoking, related diseases, and quitting smoking, starting from their first year of faculty. In this context, it is necessary to continually assess and report the smoking and depression rates of medical faculty students. With such studies, factors that influence smoking can be identified and followed, thereby allowing increased comprehension of factors that contribute to smoking in this population. In Turkey, studies concerning this important issue are insufficient and there is limited interest in creating new approaches to prevent smoking among policy makers and academics.

This study aims to investigate the frequency and characteristics of smoking among medical faculty students, assess depression symptomatology, and identify the relationships between smoking, depression, and other factors, thereby seeking to draw attention to this important issue among policy makers and academics in Turkey.

## 2. Material and Methods

### 2.1. Study Design and Ethical Approval

This descriptive cross-sectional study was conducted at Selçuk University Faculty of Medicine, Konya, Turkey, between November 2018 and February 2019. Within the scope of the “Tobacco-Free Faculty Project” initiated in this medical faculty in 2018, medical faculty students were evaluated with a smoking survey and counseling was provided to help them quit smoking. The data collected within the scope of this project were used for this study. The purposes and procedures of the study were re-appraised and approved by our ethics committee before carrying out any relevant analyses (date: 7 June 2022; no: 2022/282). All aims and means of the study were confirmed to be designed according to the ethical standards of the institutional research committee and the Helsinki declaration and its later amendments.

Students studying at Selçuk University Faculty of Medicine in the 2018–2019 academic year were included in the study. Written informed consent had been obtained from all participants before their involvement in the project. During data collection, it was planned to include all students enrolled in the medical faculty who volunteered and signed informed consent, and, thus, a targeted sample size or group was not created. Students who were not attending their classes for any reason, those on an academic break (gap year etc.), and students who refused to participate in the study were not included in the study and related data were not collected.

At the time of the study, there was a total of 1237 students registered at Selçuk University Faculty of Medicine. Among these, 120 students were not included in the analyses because they met exclusion criteria or did not approve participation or withdrew their approval. Analyses were completed on a total of 1117 participants (90.2% of all students). Among these, 209 were first-year students (95.8% of all first-year students), 207 were second-year students (88% of all second-year students), 192 were third-year students (90.9% of all third-year students), 164 were fourth-year students (82% of all fourth-year students), 165 were fifth-year students (91.6% of all fifth-year students), and 190 were sixth-year students (93.2% of all sixth-year students).

### 2.2. Data Collection

Participants were asked to complete an anonymous, self-administered questionnaire consisting of data related to age; sex; year (in the faculty: 1 to 6 years in Turkey); marriage status; place of residence; economic status; family type; history of depression diagnosis; exercise; use of alcohol, cigarettes (smoking), drugs, electronic cigarette, hookah, cigar, pipe, and chewing tobacco; the Fagerstrom Test for Nicotine Dependence (FTND); and the Beck Depression Inventory (BDI).

Some additional information about smoking was also collected. Individuals who smoked at least 100 cigarettes throughout their lives and currently smoked at least one cigarette a day were considered active smokers. Among these, participants who purchased cigarettes (packs or loose tobacco and filters to self-roll) regularly (at least once a week) were defined as regular smokers, while those who rarely purchased their own cigarettes but smoked socially (and within 30 days) were defined as occasional smokers. The ex-smoker definition was based on having a total consumption of ≥100 cigarettes, but none in the last 30 days. Non-smokers were those with an all-time consumption of <100 cigarettes and none in the last 30 days [14].

The Fagerstrom Test for Nicotine Dependence is a test developed to determine the level of nicotine addiction, consisting of 6 questions which are graded based on responses. The Turkish validity and reliability study of FTND was conducted by Uysal et al. [14]. The final FTND scores allow the classification of nicotine dependence into five levels: very low (0 to 2 points), low (3 to 4 points), moderate (5 points), high (6 to 7 points), and very high (8 to 10 points) [15]. Due to the small number of participants with high scores, students were divided into two subgroups: those with scores below 5 points and those with 5 points and above, in order to increase the power of statistical analyses.

The Beck Depression Inventory is a questionnaire consisting of 21 items evaluating a range of symptoms often observed in depression. It is largely accepted to be a relatively accurate method of quantifying the degree of depression symptoms [16]. The final BDI score can range from 0 to 63 points. The validity and reliability study of the Turkish BDI was conducted by Hisli et al., who reported a cut-off value of 17 for the Turkish population [16]. We categorized and compared students based on the 17-point threshold (<17 versus ≥17 points).

### 2.3. Statistical Analysis

All analyses, conducted with a significance threshold of *p* < 0.05, utilized IBM SPSS for Windows, Version 25.0 (IBM Corp., Armonk, NY, USA). The normal distribution of variables was assessed through examination of histogram and Q-Q plots. Numerical values were summarized with mean ± standard deviation or median (25th percentile–75th percentile), depending on normal and non-normal distribution, respectively. Categories and groups were described with count (n) and relative frequency (%).

The Mann–Whitney U test was employed for between-group analyses of continuous variables due to non-normal distribution. For categorical variables, between-group analyses were performed via chi-square, Fisher’s exact, or Fisher–Freeman–Halton tests. Multivariable logistic regression analyses were performed to identify independent factors significantly associated with smoking and BDI score. Variables showing statistical significance in univariate analysis were incorporated into the logistic regression analysis.

## 3. Results

The median age was 22 (20–24) years and 55.86% (*n* = 624) of the participants were female. Overall, 813 (72.78%) were non-smokers, 82 (7.34%) were ex-smokers, 98 (8.77%) were occasional smokers, and 124 (11.10%) were regular smokers (active smokers = 222, 19.87%). A total of 11 (0.98%) participants used electronic cigarettes, 120 (10.74%) were hookah users, 20 (1.79%) smoked cigars, 2 (0.18%) reported pipe smoking, 4 (0.36%) used chewing tobacco, 17 (1.52%) were using drugs, and 120 (10.74%) were consuming alcohol. The mean age of first smoking of active smokers was 17.53 ± 3.06 and 92 (41.44%) of them started smoking in medical school. The median duration of smoking was 3 (1–5) years. The FTND score of 56 (25.23%) for active smokers was ≥5. While age was similar between active smokers and the non-smoker and ex-smoker group (*p* = 0.340), males were over-represented in the active smoker group (*p* < 0.001). There was no significant difference between the groups in terms of faculty grade (years) distribution (*p* = 0.397). While the percentage of active smokers living at home with friends was significantly higher, the percentage of those living in state dormitories was significantly lower (*p* = 0.002). Among active smokers, the percentages of those who described their economic situation as “good” (*p* = 0.042), those who exercised regularly (*p* < 0.001), those ever diagnosed with depression (*p* < 0.001), those who consumed alcohol (*p* < 0.001), and those who used drugs (*p* = 0.010) were significantly higher (Table 1).

The median age of those with a BDI score of ≥17 was significantly lower than those with a score of <17 (*p* = 0.002). The groups were similar in terms of sex distribution (*p* = 0.480). Among those with a BDI score of ≥17, the percentage of those staying at home with friends was significantly lower and the percentage of those staying in a state dormitory was significantly higher (*p* = 0.001). Additionally, the frequencies of non-exercising students (*p* = 0.026), those with a history of depression diagnosis (*p* < 0.001), and drug users (*p* < 0.001) were significantly higher among those with a BDI score of ≥17 (Table 2). In addition, there was a very low negative correlation between FTND and BDI scores (r = −0.139, *p* = 0.038).

Multivariable logistic regression revealed that male sex (OR: 2.388, 95% CI: 1.703–3.347, and *p* < 0.001), living at home with friends (OR: 1.666, 95% CI: 1.151–2.411, and *p* = 0.007), good economic status (OR: 1.462, 95% CI: 1.059–2.018, and *p* = 0.021), depression diagnosis at any time in life (OR: 1.992, 95% CI: 1.241–3.198, and *p* = 0.004), and alcohol use (OR: 4.392, 95% CI: 2.841–6.791, and *p* < 0.001) were independently associated with active smoking (Table 3).

Multivariable logistic regression showed that being a senior (sixth year) student (OR: 0.219, 95% CI: 0.100–0.480, and *p* < 0.001) and regular exercise (OR: 0.307, 95% CI: 0.146–0.649, and *p* = 0.002) were independently associated with having a low (<17) BDI score, whereas depression diagnosis (OR: 3.239, 95% CI: 1.985–5.287, and *p* < 0.001) and drug use (OR: 5.402, 95% CI: 1.801–16.201, and *p* = 0.003) were independently associated with having a high (≥17) BDI score (Table 3).

## 4. Discussion

In the current study, which was conducted in the medical faculty of the largest province in Turkey, the smoking rates among all faculty students were 29.4% for males, 12.3% for females, and 19.8% overall. Male sex, living at home with friends, good economic status, having been diagnosed with depression, and alcohol use were independent risk factors for active smoking. A higher grade in the medical faculty was independently associated with a lower risk of depression symptoms, while depression diagnosis at any time in life and drug use were independently associated with a higher risk of depression symptoms.

Prior studies from Turkey that were conducted between 2005 and 2023, report smoking rates among medical faculty students at 3.7% to 55.6%—with considerable variations associated with grade (year) in medical school [4,17,18,19,20,21,22,23]. In the most recent study investigating this topic in Turkey, by Fakili et al., the smoking rate of medical students was reported as 34.7% for males, 14.1% for females, and 24% overall [23]. In the study by Kutlu et al., 4504 university students selected from 17 faculties in Konya Selcuk University in the 2005–2006 academic year were examined. The smoking rate was reported as 36% [24]. Researchers from other countries have also reported smoking rates among medical school students of between 6% and 49.5% [7,9,11,25,26,27,28,29,30,31]. As confirmed by our data, smoking rates among medical school students are higher than for many countries listed above. It is likely that this situation is affected by the high smoking rates in the general population, but it is difficult to make a definitive comment since the non-medical faculty population was not included in this study.

It is interesting that smoking rates are so high among medical students, even though they know very well the harm of smoking. These results, therefore, suggest the contribution of various strong factors that lead to smoking. Identifying and eliminating the reasons that encourage smoking among medical students, the healthcare providers of the future, may be one of the earliest and, therefore, most effective interventions in the fight against smoking. In this study, male sex, living at home with friends, good economic status, having been diagnosed with depression, and alcohol use were identified as independent risk factors associated with active smoking. In a study from Turkey, Şenol and colleagues showed that the first 3 years of medical education are the riskiest periods for starting smoking and that being male, having a friend who smokes in the same environment, and having a high trait anxiety score are among the other factors that lead to this outcome [32]. In another study from Turkey, male sex, alcohol use, education status of the mother, and having a smoking-related disease in the family were listed as factors that significantly influence smoking among medical faculty students [23]. In a study from Japan, medical students were more likely to smoke if they were male, attended a private school, had smoking siblings, consumed alcohol or coffee, and reported insomnia or less than 6 h of daily sleep [28]. Chkhaidze et al. reported that the only factor associated with smoking prevalence in students was male sex; however, this is likely due to the limited number of variables included in this study [7]. A national review conducted in China reported that male sex, region of residence, and increasing years in school were associated with increased rates of smoking among medical school students. This study showed that smoking rates were significantly lower in regions where socioeconomic status and the implementation and supervision of smoking ban policies were better [9]. In a study conducted among first- and second-year medical faculty students in Saudi Arabia, the most important reasons for smoking were shown to be activities in free time, peer pressure, and relieving stress. Additionally, having a friend who smokes has been identified as a major risk factor for starting smoking. The impact of the mother is a notable factor, as it has been shown that the smoking rate was found to be higher among students whose mothers were self-employed, and lower in those whose mothers were housewives or retired [27]. In many other studies, the following are presented as the most important factors increasing the frequency of smoking among medical faculty students: advanced age, male sex, alcohol use, parents’ education status, high stress, having a smoker in the family, smoking among peers, higher grade (year) in faculty, poor academic performance, depressive symptoms, use of antidepressants or anxiolytics, social and financial problems, and underlying factors involving university life and area of residence [11,29,30,31,33]. Despite some outliers [27], many studies conducted in Turkey and other countries have shown that smoking rates among medical faculty students increase from the 1st year to the 6th year (senior year, internship) [9,19,20,21]. The lower frequency of smoking among females reflects similarities with international data, but cultural and religious reasons that disproportionately impact females may also contribute to these findings. Our study supports these results and shows that the risk is higher among males. In general, it can be said that male sex, alcohol consumption, socioeconomic status, and the presence of smoker acquaintances are the strongest potential risk factors for medical students to smoke.

The fact that medical school education is a highly challenging, stressful, and long process increases the risk of mental disorders such as depression, anxiety, and psychological stress among students, and many previous studies have shown that depression is higher for medical students than the general population [12,13,34]. In a study conducted in Turkey, it was observed that 29.3% of medical students had depressive symptoms [35]. In another study, the prevalence of depression was estimated at a striking 64.2% [36]. Another aim of the current study was to measure the severity of depression symptomatology among medical school students with the BDI scale and to determine factors that strongly impacted scores. Our results showed that years in training (higher grade) was independently associated with a lower BDI score while depression diagnosis at any time in life and drug use were independently associated with a higher BDI score. Previous studies have reported numerous factors associated with depressive symptoms among medical school students, including ethnicity, sex, relationship with peers, academic performance, low family income, external pressures, school location, financial burden, physical inactivity, being a senior student (internship), perceived negative influence of night shifts, having a non-self-determined motivational profile, and having a chronic disease [13,34,35,37,38,39]. Although no significant relationship was found between sex and BDI score in our study and an inverse relationship was found between faculty grade and BDI score, many previous studies reported a significant relationship between female sex and faculty grade and the severity of depressive symptoms. This may be related to disproportionate sex distribution among students included in the studies, the difficulty levels of medical education, and differences in career opportunities after graduation between countries (and even between provinces in the same country).

The strikingly high rates of smoking and depression symptoms within the medical student population raise significant concerns and warrant thorough examination. This phenomenon is deeply troubling as it not only affects the physical and mental well-being of future healthcare professionals but also has broader implications for public health. Firstly, the co-occurrence of smoking and depression among medical students can exacerbate existing health risks. Smoking is known to be a major contributor to various serious health conditions such as cardiovascular disease, respiratory disorders, and certain cancers. When combined with depression, which can lead to unhealthy coping mechanisms and lifestyle choices, the potential for adverse health outcomes is compounded. Moreover, the stress and pressure associated with medical school can exacerbate both smoking habits and depressive symptoms, creating a harmful cycle that negatively impacts overall health and academic performance. Beyond individual health consequences, the prevalence of smoking and depression among medical students has broader implications for patient care and healthcare delivery. Healthcare providers who smoke or struggle with depression may be less effective in promoting healthy behaviors to their patients and may even inadvertently normalize unhealthy habits. Additionally, the mental health of healthcare professionals directly influences their ability to provide quality care, affecting patient outcomes and overall healthcare system efficiency. This can be seen as an indication of lax implementation of anti-tobacco measures in higher education, the lower importance given to create policies to reduce tobacco consumption in the general population, and insufficient efforts to reduce stressors affecting students in medical schools. However, in order to effectively control smoking on a population-wide scale, there is a need for well-trained professionals who can deal with this problem. Addressing this issue requires multifaceted interventions at both the individual and institutional level. Comprehensive tobacco control measures, including smoking cessation programs tailored to medical students, are essential to reduce smoking prevalence. Similarly, providing accessible mental health resources and support systems within medical schools can help alleviate stress and prevent or manage depression among students. Furthermore, promoting a culture of wellness and resilience within the medical education system is crucial to fostering a supportive environment where students feel empowered to prioritize their mental and physical well-being. Moreover, the inclusion of smoking-related themes in the curricula of universities providing health education and devoting more resources to monitoring and early detection of medical student distress may be helpful.

## 5. Limitations

Some possible limitations of the study are as follows. Because it was a single-center study and the prevalence of smoking and depression may vary significantly regionally, its results have limited generalizability to medical students across the country or globally. The self-report and cross-sectional design of the study might have limited the establishment of the causal relationship between smoking, depression, and other factors. These may have also limited the comprehension of the temporal variation in this relationship and might have caused potential measurement errors and biases. Students’ knowledge about the harms of smoking were not measured. Since they are medical faculty students, it was assumed that their knowledge level was sufficient, but the knowledge levels of 6th grade students and 1st grade students cannot be accepted to be similar. Smoking status in the family, education, and some socioeconomic characteristics of the parents were ignored. Students were asked whether they had ever been diagnosed with depression and whether they had used antidepressants, rather than basing data on medical records. The BDI scale was applied but it is a test that measures the tendency towards depression or the severity of symptoms and cannot be used as a diagnostic test. Therefore, the frequency of depression among medical faculty students in Turkey, the factors affecting the frequency of depression, and the relationship between depression and smoking need to be investigated more comprehensively. Since a population other than medical students was not included, a comparison with the general population or other professions could not be made. The fact that the data were collected five years ago is an important limitation because there may have been significant changes in the prevalence of smoking among students during that time. Finally, mood self-assessments vary depending on the time of day [40]. However, this was not taken into account in this study.

## 6. Conclusions

Our data show that almost 20% of medical school students were active smokers (occasional or regular) and the frequency of smoking was about 2.5-fold higher among males compared to females (29.4% vs. 12.3%, respectively). Independent factors associated with smoking were identified as male sex, living at home with friends, good economic status, being diagnosed with depression, and alcohol consumption. Being a senior student and exercising regularly were associated with a decrease in depressive symptoms, while current or past depression diagnosis and drug use were associated with an increase in depression symptoms. Adequately combating modifiable risk factors can contribute significantly to both improving the health and lifestyle of future physicians, which will result in better population-wide impacts on the prevention of smoking and cessation. Reducing risk factors for depression will also lead to significant improvements in smoking rates. We hope that our study will raise awareness on the subject for policy makers and academics.

## Figures and Tables

**Table 1:** Individuals’ characteristics with regard to smoking status.

		Smoking Status		
	Total	Non-Smoker and Ex-Smoker (*n* = 895)	Active Smoker (*n* = 222)	*p*
Age, years	22 (20–24)	22 (20–24)	22 (20–24)	0.340 ^a^
Sex				
Male	493 (44.14)	348 (38.88)	145 (65.32)	<0.001 ^b^
Female	624 (55.86)	547 (61.12)	77 (34.68)	
Grade (year)				
First	209 (18.71)	171 (19.11)	38 (17.12)	0.650 ^b^
Second	207 (18.53)	160 (17.88)	47 (21.17)	
Third	192 (17.19)	158 (17.65)	34 (15.32)	
Fourth	164 (14.68)	127 (14.19)	37 (16.67)	
Fifth	165 (14.77)	136 (15.20)	29 (13.06)	
Sixth	180 (16.11)	143 (15.98)	37 (16.67)	
Marital status				
Single	1097 (98.21)	877 (97.99)	220 (99.10)	0.397 ^c^
Married	20 (1.79)	18 (2.01)	2 (0.90)	
Living place				
Home with family	379 (33.93)	306 (34.19)	73 (32.88)	0.002 ^b^
Home with friends	225 (20.14)	160 (17.88)	65 (29.28)	
Home, alone	102 (9.13)	82 (9.16)	20 (9.01)	
State dorm	299 (26.77)	255 (28.49)	44 (19.82)	
Private dorm	112 (10.03)	92 (10.28)	20 (9.01)	
Family type				
Nuclear family	948 (84.87)	762 (85.14)	186 (83.78)	0.245 ^b^
Extended family	126 (11.28)	103 (11.51)	23 (10.36)	
Broken family	29 (2.60)	19 (2.12)	10 (4.50)	
At least one of the parents dead	14 (1.25)	11 (1.23)	3 (1.35)	
Economic status				
Good	425 (38.05)	326 (36.42)	99 (44.59)	0.042 ^b^
Fair	661 (59.18)	546 (61.01)	115 (51.80)	
Poor	31 (2.78)	23 (2.57)	8 (3.60)	
Do you exercise?				
Yes, regularly	108 (9.67)	71 (7.93)	37 (16.67)	<0.001 ^b^
Yes, sometimes	434 (38.85)	351 (39.22)	83 (37.39)	
No	575 (51.48)	473 (52.85)	102 (45.95)	
Ever diagnosed with depression	118 (10.56)	80 (8.94)	38 (17.12)	<0.001 ^b^
Antidepressant use	20 (1.79)	15 (1.68)	5 (2.25)	0.572 ^c^
Alcohol use	7 (2–13)	56 (6.26)	64 (28.83)	<0.001 ^b^
Drug use	935 (83.71)	9 (1.01)	8 (3.60)	0.010 ^c^
Beck Anxiety Inventory score	108 (9.67)	7 (2–13)	7 (2–13)	0.952 ^a^
<17	434 (38.85)	745 (83.24)	190 (85.59)	0.397 ^b^
≥17	182 (16.29)	150 (16.76)	32 (14.41)	

Descriptive statistics were presented by using median (25th percentile–75th percentile) for non-normally distributed continuous variables and frequency (percentage) for categorical variables. ^a^: Mann–Whitney U test, ^b^: Chi-square test, and ^c^: Fisher’s exact test.

**Table 2:** Individuals’ characteristics with regard to Beck Depression Inventory score.

	Beck Depression Inventory Score		
	<17 (*n* = 935)	≥17 (*n* = 182)	*p*
Age, years	22 (20–24)	22 (20–23)	0.002 ^a^
Sex			
Male	417 (44.60)	76 (41.76)	0.480 ^b^
Female	518 (55.40)	106 (58.24)	
Grade (year)			
First	170 (18.18)	39 (21.43)	<0.001 ^b^
Second	167 (17.86)	40 (21.98)	
Third	161 (17.22)	31 (17.03)	
Fourth	130 (13.90)	34 (18.68)	
Fifth	135 (14.44)	30 (16.48)	
Sixth	172 (18.40)	8 (4.40)	
Marital status			
Single	916 (97.97)	181 (99.45)	0.229 ^c^
Married	19 (2.03)	1 (0.55)	
Living place			
Home with family	326 (34.87)	53 (29.12)	0.001 ^b^
Home with friends	204 (21.82)	21 (11.54)	
Home, alone	81 (8.66)	21 (11.54)	
State dorm	235 (25.13)	64 (35.16)	
Private dorm	89 (9.52)	23 (12.64)	
Family type			
Nuclear family	792 (84.71)	156 (85.71)	0.055 ^d^
Extended family	112 (11.98)	14 (7.69)	
Broken family	20 (2.14)	9 (4.95)	
At least one of the parents dead	11 (1.18)	3 (1.65)	
Economic status			
Good	365 (39.04)	60 (32.97)	0.227 ^b^
Fair	546 (58.40)	115 (63.19)	
Poor	24 (2.57)	7 (3.85)	
Smoking status			
Non-smoker	680 (72.73)	133 (73.08)	0.381 ^b^
Ex-smoker	65 (6.95)	17 (9.34)	
Smoker, occasionally	87 (9.30)	11 (6.04)	
Smoker, regularly	103 (11.02)	21 (11.54)	
Do you exercise?			
Yes, regularly	98 (10.48)	10 (5.49)	0.026 ^b^
Yes, sometimes	370 (39.57)	64 (35.16)	
No	467 (49.95)	108 (59.34)	
Ever diagnosed with depression	82 (8.77)	36 (19.78)	<0.001 ^b^
Antidepressant use	15 (1.60)	5 (2.75)	0.353 ^c^
Alcohol use	100 (10.70)	20 (10.99)	1.000 ^b^
Drug use	8 (0.86)	9 (4.95)	<0.001 ^c^

Descriptive statistics were presented by using median (25th percentile–75th percentile) for non-normally distributed continuous variables and frequency (percentage) for categorical variables. ^a^: Mann–Whitney U test, ^b^: Chi-square test, ^c^: Fisher’s exact test, and ^d^: Fisher–Freeman–Halton test.

**Table 3:** Significant factors independently associated with active smoking and high (≥17) BDI score: multivariable logistic regression.

	β Coefficient	Standard Error	*p*	Exp (β)	95.0% CI for Exp (β)	
Active smoking						
Sex, Male	0.870	0.172	<0.001	2.388	1.703	3.347
Living at home with friends	0.510	0.189	0.007	1.666	1.151	2.411
Good economic status	0.380	0.165	0.021	1.462	1.059	2.018
Exercise regularly	0.402	0.247	0.103	1.495	0.922	2.424
Ever diagnosed with depression	0.689	0.242	0.004	1.992	1.241	3.198
Alcohol use	1.480	0.222	<0.001	4.392	2.841	6.791
Drug use	0.047	0.581	0.935	1.049	0.336	3.275
Constant	−2.478	0.160	<0.001	0.084		
High (≥17) BDI score						
Age	−0.060	0.044	0.168	0.942	0.864	1.026
Year in medical school (Sixth)	−1.519	0.400	<0.001	0.219	0.100	0.480
Living at home with friends	−0.432	0.262	0.099	0.649	0.389	1.084
Exercise regularly	−1.179	0.381	0.002	0.307	0.146	0.649
Ever diagnosed with depression	1.175	0.250	<0.001	3.239	1.985	5.287
Drug use	1.687	0.560	0.003	5.402	1.801	16.201
Constant	−0.205	0.930	0.826	0.815		

Nagelkerke R^2^ = 0.176 for active smoking and 0.111 for high (≥17) Beck Depression Inventory score, BDI: Beck Depression Inventory, and CI: confidence interval.

## Data Availability

The data presented in this study are available on request from the corresponding author.
